# Comparison of a Urine Antigen Assay and Multiple Examinations with the Formalin-Ethyl Acetate Concentration Technique for Diagnosis of Opisthorchiasis

**DOI:** 10.4269/ajtmh.23-0132

**Published:** 2023-05-22

**Authors:** Chanika Worasith, Anchalee Techasen, Kunyarat Duenngai, Kitti Intuyod, Kulthida Y. Kopolrat, Jiraporn Sithithaworn, Watcharin Loilome, Thomas Crellen, Melissa R. Haswell, Paiboon Sithithaworn

**Affiliations:** ^1^ Department of Parasitology, Faculty of Medicine, Khon Kaen University, Khon Kaen, Thailand;; ^2^ Cholangiocarcinoma Research Institute, Khon Kaen University, Khon Kaen, Thailand;; ^3^ Faculty of Associated Medical Sciences, Khon Kaen University, Khon Kaen, Thailand;; ^4^ Department of Thai Traditional Medicine, Faculty of Science and Technology, Phetchabun Rajabhat University, Phetchabun, Thailand;; ^5^ Department of Pathology, Faculty of Medicine, Khon Kaen University, Khon Kaen, Thailand;; ^6^ Faculty of Public Health, Kasetsart University Chalermphrakiat Sakon Nakhon Province Campus, Sakon Nakhon, Thailand;; ^7^ Department of Biochemistry, Faculty of Medicine, Khon Kaen University, Khon Kaen, Thailand;; ^8^ School of Biodiversity, One Health and Veterinary Medicine, University of Glasgow, Glasgow, United Kingdom;; ^9^ Big Data Institute, Nuffield Department of Medicine, University of Oxford, Oxford, United Kingdom;; ^10^ Indigenous Strategy and Services, University of Sydney, Sydney, Australia;; ^11^ School of Geosciences, University of Sydney, Sydney, Australia;; ^12^ School of Public Health and Social Work, Queensland University of Technology, Brisbane, Australia

## Abstract

Detection of worm antigen in urine is a sensitive diagnostic method for opisthorchiasis, particularly for light-intensity infections; however, the presence of eggs in feces is essential for validating results from the antigen assay. To address the issue of low sensitivity of fecal examination, we modified the protocol for the formalin-ethyl acetate concentration technique (FECT) and compared it against urine antigen measurements for detection of the parasite *Opisthorchis viverrini*. First, we optimized the FECT protocol by increasing the number of drops for examinations from the standard two drops to a maximum of eight. We were able to detect additional cases after examination of ≥ 3 drops, and the prevalence of *O. viverrini* saturated after examination of ≥ 5 drops. We then compared the optimized FECT protocol (examining five drops of suspension) against urine antigen detection for the diagnosis of opisthorchiasis in field-collected samples. The optimized FECT protocol detected *O. viverrini* eggs in 25 of 82 individuals (30.5%) who had positive urine antigen tests but were fecal egg negative by the standard FECT protocol. The optimized protocol also retrieved *O. viverrini* eggs in 2 of 80 antigen-negative cases (2.5%). In comparison with the composite reference standard (combined FECT and urine antigen detection), the diagnostic sensitivity of examining two and five drops of FECT and the urine assay was 58.2, 67, and 98.8%, respectively. Our results show that multiple examinations of fecal sediment increase the diagnostic sensitivity of FECT and thus provide further support for the reliability and utility of the antigen assay for diagnosis and screening of opisthorchiasis.

## INTRODUCTION

Infection with the liver fluke *Opisthorchis viverrini* remains a serious health problem in Southeast Asia. The resulting disease, opisthorchiasis, is considered a neglected tropical disease under the grouping of food-borne trematodiasis.^1^ *Opisthorchis viverrini* together with *Clonorchis sinensis* have been classified as group 1 carcinogens with infection-causing bile duct cancer in humans (cholangiocarcinoma; CCA).^2^^,^^3^ With the highly effective drug praziquantel widely available, the fundamental approach to combat CCA in endemic areas requires a comprehensive public health campaign that includes the control and elimination of *O. viverrini* and its carcinogenic relative, *C. sinensis*.^4^^,^^5^ To achieve such a goal, a robust and practical diagnostic tool for improved screening of *O. viverrini* and *C. sinensis* in endemic areas is needed.

Definitive diagnosis of *O. viverrini* infection has to date depended on conventional parasitological methods to detect eggs in feces, the most sensitive of which is the formalin-ethyl acetate concentration technique (FECT).^6^^,^^7^ Currently, these methods are unreliable at light intensities (fewer than 20 worms) of infection^8^^,^^9^ and have several drawbacks. First, egg detection in stools requires the extensive time of experienced microscopists to detect and distinguish *O. viverrini* eggs from those of coendemic minute intestinal flukes.^10^ Further, fecal eggs may be absent in prepatent infection and when egg passage is obstructed by chronic fibrosis or by dead adult worms after recent anthelmintic treatment.^6^^,^^11^^,^^12^ Repeated stool examination is recommended to increase the reliability of fecal examination,^13^^,^^14^ but this increases the logistical complexity, cost, and human resources of population screening.

Among the available methods, molecular diagnosis helps to overcome the difficulty of differentiating eggs of *O. viverrini* from those of other fish-borne trematodes.^6^^,^^11^^,^^15^ For antigen detection, there have been limited reports on coproantigen detection^16^^,^^17^ and more recently with greater sensitivity.^18^ Subsequently, a novel antigen detection method in urine was established for diagnosis of human opisthorchiasis that allows more convenient sample collection and apparently higher diagnostic accuracy than fecal examination.^19^^,^^20^ In comparison with fecal examination by FECT, urine antigen detection gave a similar detection rate in a high-transmission area but it was more sensitive in a low-transmission area.^20^ We hypothesize that examining additional drops of FECT-processed fecal sediment would be the least laborious method of conducting repeat stool examinations; however, no systematic assessment of the sensitivity of multiple-drop examination in relation to the urine antigen assay in opisthorchiasis has been published.

The aim of this study is to improve the sensitivity of fecal examination by FECT and its relationship with urine antigen detection in opisthorchiasis. First, we determined the optimal FECT examination by increasing the number of drops of fecal sediment for examination to maximize the positive detection rate. We then validated the optimized FECT protocol in a cross-sectional survey within an endemic community for opisthorchiasis in northeast Thailand by assessing qualitative and quantitative agreement with the urine antigen assay. Our results provide further evidence of the reliability of the urine antigen assay for diagnosis and population screening of opisthorchiasis.

## MATERIALS AND METHODS

### Study area and study design.

For the optimization study phase, the clinical samples were drawn from a project on the morbidity of opisthorchiasis that originated from communities in northeast Thailand.^20^ The aim of this study was to improve the sensitivity of FECT by increasing the number of drops of fecal sediment for examination from two to eight drops. The analysis was performed on stool samples that were egg negative by initial FECT screening but whose paired urine sample was urine antigen positive. In the validation study phase, the samples were from a cross-sectional epidemiological survey of *O. viverrini* infection between January and December 2018 in Na Mon District, Kalasin Province, a known endemic area^7^ in northeast Thailand. The optimized FECT protocol (examination of five drops) was applied to field-collected samples and compared with the urine antigen assay to assess the diagnostic accuracy for opisthorchiasis.

### Fecal examination by the formalin-ethyl acetate concentration method.

Fecal samples were processed by experienced laboratory technicians as described previously.^21^ Two grams of fresh stool was fixed with 10% formalin and thoroughly shaken before being strained through gauze. Three milliliters of ethyl acetate was added to the mixture to extract fat from the feces. After vigorous shaking and centrifugation at 2,500 rpm (769 × *g*) for 5 minutes, the supernatant was discarded and the remaining matter was resuspended in 10% formalin. A drop of fecal suspension was placed on a slide and examined. This was done in duplicate as the standard protocol (two-drop examination) and increased to five drops in the optimized protocol and was used for the examination and enumeration of parasites. The intensity of infection was calculated by the number of eggs counted per drop examined divided by 2 (grams of stool) and multiplied by the total drops (volume) of fecal suspension.

### Determination of urine *O. viverrini* antigen by a monoclonal antibody-based ELISA.

First morning midstream urine samples were collected in containers and kept in an ice box during transport to the laboratory. Urine samples were centrifuged at 1,500 rpm (277 × *g*) at 4°C for 15 minutes and the clarified supernatants were aliquoted and stored at −20°C until used in the urine monoclonal antibody-based ELISA. Urine samples were pretreated with an equal volume of 4% trichloroacetic acid (TCA) solution, incubated for 20 minutes at room temperature, and then neutralized with an equal volume of 0.244 M carbonate buffer (pH 9.6).^19^^,^^20^

The protocol for antigen detection in urine was performed as previously described.^19^ Briefly, polystyrene microtitration plates (Nunc, Roskilde, Denmark) were coated with 5 μg/mL of the monoclonal antibody diluted in 50 mM bicarbonate buffer (pH 9.6). The plates were sealed and incubated overnight at 4°C and on the following day were washed three times with 0.05% Tween 20 in PBS (pH 7.4; PBST). The plates were blocked with 200 μL of 5% skimmed milk powder in PBST, incubated at 37°C for 1 hour, and washed again three times with PBST. Urine samples treated with TCA (100 μL per well) were added to the wells in duplicate and incubated at 37°C for 2 hours. The plates were washed five times with PBST, and 100 μL of a protein A-purified rabbit IgG (10 μg/mL) against crude *O. viverrini* antigen extract was added to each well and incubated at 37°C for 2 hours. After three washes, 1:4,000 diluted biotinylated goat anti-rabbit IgG (Invitrogen, Carlsbad, CA) in PBST was added and incubated at 37°C for 1 hour. Subsequently, the plates were washed three times with PBST, and 100 μL per well of streptavidin–horseradish peroxidase conjugate (GE Healthcare, Buckinghamshire, United Kingdom) diluted 1:5,000 in PBST was added. After incubation for 30 minutes, the plates were washed three times with PBST, and the substrate solution *O*-phenylenediamine hydrochloride (Sigma, St. Louis, MO) was added to the wells and incubated for 20 minutes in a dark box at room temperature. The reaction was stopped by the addition of 2 M sulfuric acid (H_2_SO_4_), and the plates were read on an absorbance reader (Tecan, Grödig, Austria) at the optical density (OD) of 492 nm.

The OD values were transformed into concentrations of *O. viverrini* antigens in urine using standard curves and then expressed as ng/mL. A sample was considered positive when antigen concentration was > 19.4 ng/mL.^19^^,^^20^

### Data analysis and statistical methods.

Data were entered into a spreadsheet using the Excel program (Microsoft, Redmond, WA) and statistically analyzed using SPSS version 22 (IBM, Armonk, NY). The threshold for statistical significance was set at *P* < 0.05.

The intensity of infection was expressed as eggs per gram of feces (EPG) obtained from FECT examinations and categorized as negative (EPG = 0), mild- (EPG = 1–50), moderate- (EPG = 51–100), and heavy-infection cases (EPG > 100).^19^^,^^20^ For the urine antigen assay, the intensity of infection was based on the levels of antigen in the urine and was categorized as negative (antigen concentration < 19.4 ng/mL), low positive (19.4–30.0 ng/mL), moderate positive (30.1–50.0 ng/mL), and high positive (>50.0 ng/mL).

Cumulative positive rates were calculated for FECT, and the McNemar χ^2^ test was used to compare the prevalence of *O. viverrini* infection between the varying FECT optimization protocols. The correlation between fecal egg count and urine antigen concentration was determined by Spearman’s correlation test. Diagnostic accuracy in terms of sensitivity, specificity, positive predictive value (PPV), and negative predictive value (NPV) was estimated by receiver operating characteristic curve analysis using MedCalc (MedCalc Software, Ostend, Belgium).

## RESULTS

### Phase 1: optimization study.

#### Multiple examinations.

Among the 50 samples found initially to be antigen positive but egg negative by the standard FECT, 17 (34%) were found to be egg positive with examination of the maximum eight drops of sediment (Table 1). Although no eggs were detected by examination of a second drop (0% egg positive), cumulative egg detection rates increased from 10 to 22% with examination of three to eight drops. The cumulative positive rate for eight drops (17/50; 34%) was significantly higher than for three drops (5/50; 10%) (*P* < 0.001) and four drops (9/50; 18%) (*P* < 0.01). However, no significant difference in egg positivity was seen between examinations of five, six, and seven drops compared with eight drops (McNemar χ^2^ test; *P* > 0.05). Estimates of the intensity of infection (EPG) were similar between three and eight drops (mean 4.7; SD 2.4; range 1–26). Therefore, the optimal protocol for FECT was determined as examination of five drops.

**Table 1:** The detection rate of *O. viverrini* eggs assessed by multiple examination of FECT sediment (two to eight drops), cumulative positive rates, and fecal egg counts (EPG) in individuals with positive urine antigen tests but fecal egg negative by standard FECT (*N* = 50)

Drops examined	No. of *O. viverrini* egg positive (*n*)	No. of cumulative egg positive (%)	EPG (mean ± SD)
2	0	0 (0)	0
3	5	5 (10)	3.2 ± 1.1
4	4	9 (18)	5.5 ± 2.1
5	6	15 (30)	4.8 ± 2.4
6	1	16 (32)	7.9 ± 2.3
7	1	17 (34)	3.6 ± 3.0
8	0	17 (34)	4.3 ± 2.4

EPG = egg per gram feces; FECT = formalin-ethyl acetate concentration technique.

### Phase 2: validation study.

#### Characteristics of study participants.

**Figure 1.**
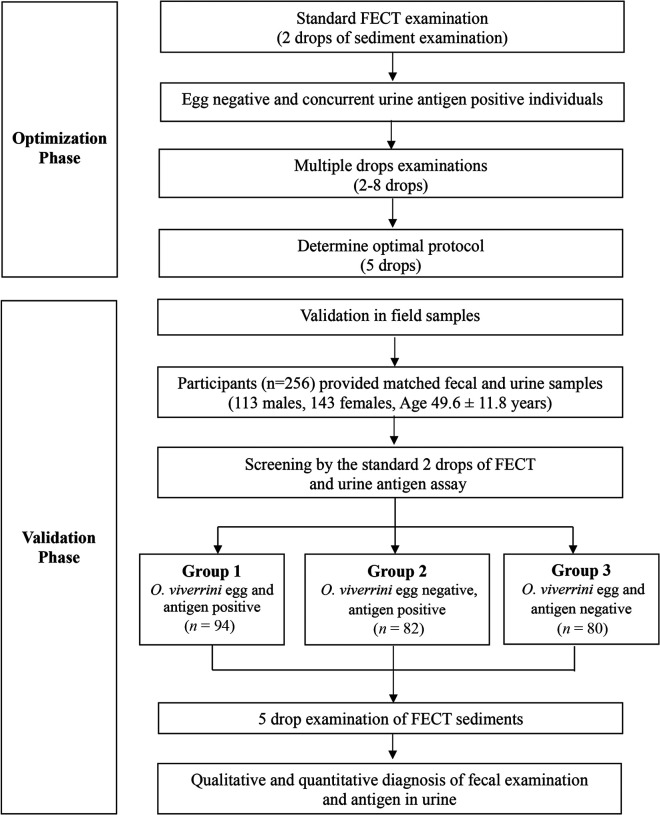
Study design. Optimization (phase 1) was designed to adjust the number of drops for FECT examination in urine antigen-positive individuals. Validation (phase 2) applied the optimized FECT protocol to field-collected samples from an endemic area of opisthorchiasis to compare with results from the urine antigen test. FECT = formalin-ethyl acetate concentration technique.

Initially, 675 individuals were invited to participate in this study; a subset of 256 individuals met the inclusion criteria and provided complete paired feces and urine specimens for laboratory analysis in the study (Figure 1). The average age of the participants was 49.6 ± 11.8 years (range 17–68 years), which included 113 males and 143 females. Based on the standard FECT protocol for parasitic infection (examination of two drops), the participants were separated into three groups: *O. viverrini* egg and antigen positive (group 1; *N* = 94), *O. viverrini* egg negative but antigen positive (group 2; *N* = 82), and *O. viverrini* egg and antigen negative (group 3; *N* = 80). The standard protocol (two drops) and optimized protocol of five drops of sediment were used for fecal examination.

#### Qualitative diagnosis between two- and five-drop examination.

Group 1 included 94 out of 256 individuals (36.7%) tested positive for *O. viverrini* by both by antigen detection and egg detection from examination of two or more drops of FECT sediment (Table 2). In groups 2 and 3, all individuals (100%) were egg negative by two drops, whereas 25 out of 82 cases (30.5%) in group 2 (urine antigen positive) and two cases (2.5%) in group 3 (urine antigen negative) were egg positive by five-drop examination. The overall prevalence of *O. viverrini* by five drops (47.2%) was significantly higher than the two-drop protocol (36.7%) (McNemar test; *P* < 0.05).

**Table 2:** Detection rates of *O. viverrini* eggs by the standard two-drop and optimized five-drop examination protocol of FECT sediments in diagnostic groups based on the urine antigen assay (*N* = 256)

Group	*n*	Antigen concentration (mean ± SD), ng/mL	Urine antigen assay results	No. of *O. viverrini* positive by FECT protocol (%)	
				Standard two drops	Optimized five drops
1	94	87.1 ± 99.1	Positive	94 (100)	94 (100)
2	82	29.5 ± 7.5	Positive	0 (0)	25 (30.5)
3	80	14.2 ± 1.8	Negative	0 (0)	2 (2.5)

FECT = formalin-ethyl acetate concentration technique.

Table 3 reports mixed infections between *O. viverrini* and other helminths, including minute intestinal flukes, *Strongyloides stercoralis*, and *Echinostoma* spp., found by five-drop examination, but none were detected by two-drop examination.

**Table 3:** Detection of other helminth infections concurrently found with *O. viverrini* by the standard two-drop and modified five-drop FECT examination among 256 individuals

Parasitic infection	No. of positive (%)	
	Two drops	Five drops
Minute intestinal fluke	0 (0)	15 (5.9)
*S. stercoralis*	0 (0)	6 (2.3)
*Echinostoma* spp.	0 (0)	1 (0.4)

FECT = formalin-ethyl acetate concentration technique.

#### Diagnostic accuracy.

Analysis of samples from fecal egg count-positive individuals (*N* = 121) by examining five drops of FECT sediment had a significantly higher sensitivity of 97.6% compared with 75.2% sensitivity by the standard two-drop examination (*P* < 0.001). The NPVs were 64.6 and 88.3% by two and five drops of FECT sediment, respectively.

When the combined results from five drops of fecal sediment and the urine antigen assay as a composite reference standard method were used, the diagnostic sensitivity of five-drop examination (68.0%) was significantly higher than the standard two drops (52.8%) (*P* < 0.001) (Table 4). The urine antigen assay provided the highest sensitivity (98.8%) among the three methods. The specificities and PPVs were 100% for all methods.

**Table 4:** Diagnostic performance analysis of the standard two-drop and five-drop examination protocols of fecal sediment and the urine antigen assay for diagnosis of *O. viverrini* infection (*N* = 256)

	Composite FECT and urine antigen assay*			
Diagnostic method	Sensitivity, % (95% CI)	Specificity, % (95% CI)	PPV, % (95% CI)	NPV, % (95% CI)
Two-drop FECT	52.8 (0.45–0.60)	100 (0.94–1.00)	100 (0.95–1.00)	48.1 (0.40–0.56)
Five-drop FECT	68.0 (0.60–0.75)	100 (0.94–1.00)	100 (0.96–1.00)	57.8 (0.49–0.66)
Urine antigen assay	98.8 (0.95–0.99)	100 (0.94–1.00)	100 (0.97–1.00)	97.5 (0.90–0.99)

FECT = formalin-ethyl acetate concentration technique; NPV = negative predictive value; PPV = positive predictive value.

* Using the combined results of five-drop examination of fecal sediment and the urine antigen assay as a composite gold standard.

#### Quantitative relationship between fecal egg count and urine antigen concentration.

Comparisons of fecal egg counts by two- and five-drop examinations of FECT according to intensity groups based on *O. viverrini* antigen concentration in urine are shown in Table 5. To explore this, urine samples were divided into groups, namely negative, low, moderate, and high antigen concentration. By FECT examination using five drops, the subjects in the negative, low positive, and moderate positive groups had comparable numbers of *O. viverrini* EPG that were obtained by two-drop examination. The highest EPGs for both two- and five-drop examination of FECT were in the high antigen concentration group.

**Table 5:** Relationship between the fecal egg count of *O. viverrini* (EPG) by two- and five-drop examination protocols of fecal sediment by FECT and the intensity class determined by urine antigen levels (*N* = 256)

Standard FECT	Antigen level*	*N*	Geometric mean, EPG ± SE (range)	
			Two drops	Five drops
Negative	Negative	80	0 (0)	2.1 ± 1.1 (2.0–2.2)
	Low	52	0 (0)	4.2 ± 2.0 (1.5–21)
	Moderate	30	0 (0)	4.1 ± 2.3 (1.2–12)
	High	–	–	–
Positive	Low	24	29.2 ± 2.2 (8.8–113.9)	24.9 ± 2.0 (5.3–72.9)
	Moderate	22	42.5 ± 1.8 (15.3–134.3)	43.1 ± 1.6 (20.3–146.3)
	High	48	73.5 ± 2.3 (24.2–634.1)	89.9 ± 2.1 (32.2–659.6)

EPG = egg per gram feces; FECT = formalin-ethyl acetate concentration technique.

* Negative: antigen concentration < 19.4 ng/mL; low: antigen concentration 19.4–30.0 ng/mL; moderate: antigen concentration 30.1–50.0 ng/mL; and high: antigen concentration > 50.0 ng/mL.

Although the EPG of individuals who were identified as egg positive only when five drops were examined was very low regardless of urine antigen level, there was a clear positive relationship between the EPG determined by both two- and five-drop examinations and groups stratified by urine antigen level.

**Figure 2.**
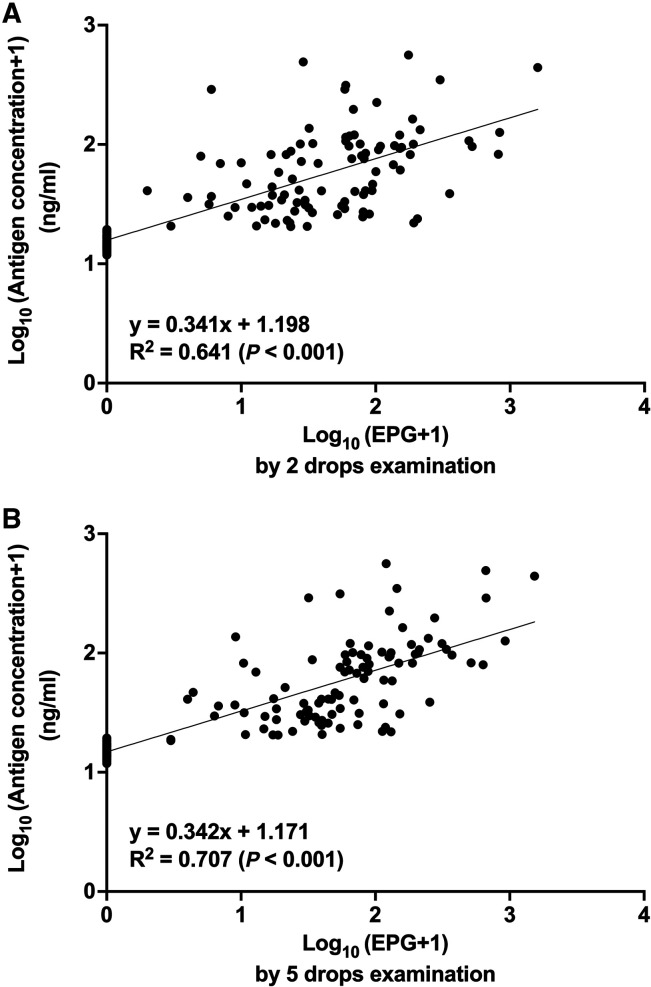
The correlation between *O. viverrini* antigen concentration (log-transformed values) determined by urine antigen assay and EPG (log-transformed values), calculated by using two-drop (**A**) and five-drop examinations (**B**) of the fecal sediments prepared by FECT from Na Mon District, Kalasin Province (*N* = 256). EPG = egg per gram feces; FECT = formalin-ethyl acetate concentration technique.

Linear regression further confirmed this relationship, with strong correlations shown between log-transformed concentration of worm antigen in urine and log-transformed EPG using two-drop (*R*^2^ = 0.641; *P* < 0.001; Figure 2A) and five-drop examination protocols of fecal sediment by FECT (*R*^2^ = 0.701; *P* < 0.001; Figure 2B).

## DISCUSSION

To maximize the sensitivity of fecal egg detection, we performed multiple examinations of fecal sediment to increase the probability of detecting *O. viverrini* eggs in feces should they exist in antigen-positive individuals who are fecal egg negative by the standard FECT protocol. The examinations of FECT sediment with two to eight drops showed more positive cases detected by examining a third drop, and a stable prevalence of *O. viverrini* was attained beginning at five drops onward. The optimized FECT protocol using five drops found additional egg-positive cases that were negative by the standard two-drop protocol. The optimized protocol yielded fecal egg counts that significantly correlated with antigen concentration of *O. viverrini* in urine and discovered additional parasitic coinfections that were missed by the standard two-drop protocol.

In the optimization study, increased sediment examination elevated the sensitivity of FECT in urine antigen-positive cases and also showed a low-grade fecal egg count (mean EPG < 7.9). The observed data for egg detection and fecal egg count suggested that multiple-drop examination of a single fecal sample partially validated the result of urine antigen tests. It remains to be seen whether or not multiple-day examination (i.e., 3 days) can further improve the egg detection rate in urine antigen-positive opisthorchiasis.^14^

In the validation study, the prevalence of *O. viverrini* detected by the modified five-drop protocol was significantly higher than the standard two-drop FECT protocol. When considering only antigen-positive individuals, 30.5% were egg positive. In the egg- and antigen-negative group (group 3), a small proportion of individuals (2.5%) were egg positive. The underdiagnosis of antigen detection in this particular group (group 3) deserves further investigation (i.e., by multiple-day examinations of urine samples). The other advantage of the modified five-drop protocol was that it yielded additional coexisting parasitic infections, including minute intestinal fluke, *Strongyloides stercoralis*, and *Echinostoma* spp. in addition to *O. viverrini.* It can be anticipated that multiple-sample examinations will reveal concurrent infection with other helminthiasis.

For diagnostic performance analysis, the modified FECT protocol (five-drop examination) had increased sensitivity compared with the standard FECT protocol (two-drop examination). Compared with the composite reference standard (combined fecal examination and urine assay), urine antigen detection had 98.8% sensitivity with 52.8 and 68% for the two- and five-drop protocols, respectively. It had been suggested that 3 consecutive days of stool examinations yields higher accuracy for diagnosis of *O. viverrini* and other helminth infections.^13^ However, multiple fecal collections over more than 1 day is impractical and imposes an additional workload on volunteers and public health workers. Alternatively, increasing the number of drops examined by FECT prepared from a single stool sample can improve the diagnostic sensitivity of *O. viverrini* infection with significantly less additional resources. For quantification of *O. viverrini* eggs, there were similar correlations between fecal egg count (both two and five drops) and antigen concentration in urine (low to high antigen, 19.4 to > 50 ng/mL urine). However, in egg-negative individuals by standard FECT (two drops), the modified protocol (five drops) yielded lower mean egg counts (EPG = 4) in the low- and moderate-antigen groups (19.4–50 ng/mL urine). The data for inconsistency in EPG (differing by 10- to 20-fold) in relation to antigen concentration (i.e., low, moderate, and high) are puzzling and require further study. In previous studies, we reported that urine as well as coproantigens were quantitatively correlated with fecal egg counts in two endemic communities with opisthorchiasis.^19^^,^^20^ There is a need to investigate the correlation patterns between urine antigen and fecal egg count in other endemic settings.

We previously reported that antigen detection in urine was more sensitive than fecal examination for diagnosis of opisthorchiasis (i.e., 17–44% of positive antigen tests were found in individuals with no observed fecal eggs).^14^^,^^19^^,^^20^ Persistency of antigen in urine over a period of 10 months indicated the reliability of antigen detection in diagnosis of opisthorchiasis.^22^ In this study, based on the standard FECT protocol (two-drop examination), egg detections were negative in 57 of 176 individuals (32.3%) in antigen-positive groups (groups 1 and 2). Within *O. viverrini* egg-negative but antigen-positive individuals, the positive rates of *O. viverrini* in both the optimization and validation studies were similar (about 30%) by the optimized FECT protocol. Hence, there were considerable numbers of individuals who were antigen positive but fecal egg negative. There are many supporting lines of evidence to suggest that these observations are not caused by false positive urine antigen tests. First, the results from a separate diagnostic coproantigen test have been shown to correlate with urine antigen tests and detected cases in a similar proportion as detected in fecal egg-negative individuals.^20^ Second, praziquantel treatment in urine antigen-positive but fecal egg-negative individuals resulted in a reduction of antigen levels in urine, and these individuals became antigen negative at 4 weeks posttreatment.^23^ This report suggests the role of adult worms, rather than eggs, as the source of the excretory-secretory antigen detected in urine in opisthorchiasis-positive individuals. A similar situation was previously observed in an autopsy study in which eggs in feces were not found in the majority of individuals with < 20 adult worms.^8^ Moreover, a laboratory animal study showed that urine and coproantigen levels corresponded to the presence of worms in the liver of *O*. *viverrini*-infected hamsters.^12^ Although the nature of the antigen(s) detected in urine and feces is currently unknown and being investigated, based on the above evidence in human and experimental opisthorchiasis, we argue that positive antigen detection in urine in fecal egg-negative individuals represents active *O*. *viverrini* infection. In addition to qualitative diagnosis of opisthorchiasis, the urine antigen assay can also be used quantitatively to assess the outcomes of anthelmintic treatment of individual patients or as part of a control program. Because of its ease of use and noninvasive urine sample collection, the urine antigen assay offers the potential to revolutionize the diagnosis of liver fluke infection and provide an effective tool for control and elimination of these tumorigenic helminths.

There are several limitations to our study. First, the modified protocol of multiple examinations relied on a single fecal sample; the impacts of multiple sample examination as well as daily variation of fecal examination were not investigated. Second, because the antigen concentration in feces is higher than in urine,^18^^,^^20^ additional coproantigen detection should be performed for comparison between egg-positive cases and the egg-negative cases who had positive urine antigen tests. Lastly, because we used only FECT in this study, whether multiple replicate examinations by other methods such as the Kato–Katz method would yield similar results is unknown. Nevertheless, based on the modified protocol of FECT examination in this study, we demonstrate egg-positive detection in cases that were negative by the standard FECT protocol; however, the modified FECT technique remains less sensitive than the urine antigen test for screening of opisthorchiasis.

## CONCLUSION

The optimized five-drop protocol using a single fecal sample had greater sensitivity than the standard two-drop FECT protocol (i.e., 68 and 52%, respectively). The optimized protocol detected *O. viverrini* eggs in 30% of urine antigen-positive individuals who had initially tested negative for fecal eggs by the standard protocol. Moreover, egg-positive cases (2.5%) and parasitic coinfections were uncovered by the optimized five-drop FECT protocol. The concordance of egg-positive detection and quantification with the urine antigen assay for opisthorchiasis provides further support for the reliability and utility of the urine assay for diagnosis and screening of opisthorchiasis.
